# Anxiety and Depression in Patients with Pulmonary Arterial Hypertension and Chronic Thromboembolic Pulmonary Hypertension after the Removal of COVID-19 Pandemic Restrictions

**DOI:** 10.3390/jcm13123532

**Published:** 2024-06-17

**Authors:** Maria Wieteska-Miłek, Anna Witowicz, Sebastian Szmit, Michał Florczyk, Michał Peller, Milena Dzienisiewicz, Marcin Kurzyna

**Affiliations:** 1 Department of Pulmonary Circulation, Thromboembolic Diseases and Cardiology, Centre of Postgraduate Medical Education, European Health Centre, ERN-LUNG Member, ul. Borowa 14/18, 05-400 Otwock, Poland; awitowicz@wim.mil.pl (A.W.); michal_florczyk@wp.pl (M.F.); marcin.kurzyna@ecz-otwock.pl (M.K.); 2 Department of Cardiology and Internal Diseases, Military Institute of Medicine-National Research Institute, 04-141 Warsaw, Poland; 3 Department of Cardio-Oncology, Centre of Postgraduate Medical Education, 01-813 Warsaw, Poland; s.szmit@gmail.com; 4 1st Department of Cardiology, Medical University of Warsaw, 02-091 Warszawa, Poland; michalpeller@gmail.com; 5 European Health Centre, 05-400 Otwock, Poland; milena.dzienisiewicz@gmail.com

**Keywords:** fear of COVID-19, pandemic, anxiety, depression, HADS scale, pulmonary arterial hypertension, chronic thromboembolic pulmonary hypertension

## Abstract

**Background:** There was increased risk of mental disturbances during the COVID-19 pandemic. Patients with chronic diseases, including pulmonary arterial hypertension (PAH) and chronic thromboembolic pulmonary hypertension (CTEPH), were particularly vulnerable. Our previous study showed high levels of fear of COVID-19 (FCV-19S), anxiety (HADS-A), and depression (HADS-D) in the second year of the pandemic among PAH/CTEPH patients. The aim of the present study was to assess changes in the levels of FCV-19S, HADS-A, and HADS-D after removing restrictions related to the COVID-19 pandemic. **Methods:** In this prospective, single-center study, 141 patients (62% females, 64% PAH) with a median age of 60 (range 42–72) years were included. Patients completed appropriate surveys in the second year of the pandemic, and then, after the restrictions were lifted in Poland (after 28 March 2022). **Results:** FVC-19S decreased significantly from 18 (12–23) to 14 (9–21), *p* < 0.001. The levels of anxiety (HADS-A ≥ 8 points) and depression (HADS-D ≥ 8 points) were abnormal in 26% and 16% of patients, respectively; these did not change at follow-up (*p* = 0.34 for HADS-A and *p* = 0.39 for HADS-D). **Conclusions**: Among PAH/CTEPH patients, fear of COVID-19 decreased significantly after the COVID-19 pandemic restrictions were removed, but anxiety and depression remained high, indicating that the COVID-19 pandemic was not a major factor in causing these disorders.

## 1. Introduction

The COVID-19 pandemic has impacted the physical and mental health of people worldwide [1]. The pandemic increased the risk of death due to the severe course of SARS-CoV-2 infection, increased risk of isolation, forced quarantine, difficulties in accessing appropriate medical care, and heightened levels of overall stress, insomnia, anxiety, and depression [1]. Patients with chronic diseases such as pulmonary arterial hypertension (PAH) and chronic thromboembolic pulmonary hypertension (CTEPH) were particularly vulnerable [2,3].

PAH and CTEPH are rare, chronic diseases characterized by precapillary pulmonary hypertension (PH), confirmed by right heart catheterization [4,5]. PAH is diagnosed in the absence of other defined causes of precapillary PH, while CTEPH is diagnosed when pulmonary hypertension develops due to the obstruction and remodeling of the pulmonary artery by thromboembolic material [4,5]. Patients with PAH are treated with specific drugs that dilate the pulmonary arterioles and reduce pulmonary vascular resistance [4,5]. CTEPH patients are additionally treated with pulmonary endarterectomy or balloon pulmonary angioplasty [4,5,6,7,8]. The progression of the disease, suboptimal management, and infections can cause exacerbations of right heart failure and potentially lead to death [6,9,10].

PAH/CTEPH patients with long-term, life-threatening diseases are particularly susceptible to mental disorders. According to various sources, before the COVID-19 pandemic, the prevalence of anxiety and depression in these patient populations ranged from 20% to 50% and from 8% to 53%, respectively [11,12,13]. Some studies have reported a high prevalence of mental disorders in patients with PAH or CTEPH in the initial phase of the pandemic [2,3,14]. However, none of these studies assessed whether lifting COVID-19-related restrictions impacted the prevalence of anxiety–depressive disorders in these patients, or compared the same patient population before and during the pandemic.

In this prospective, single-center study, we examined the prevalence of the fear of COVID-19, anxiety, and depression in PAH/CTEPH patients, using structured clinical scales, during the second year of the pandemic and following the lifting of COVID-19 related restrictions in Poland.

## 2. Materials and Methods

### 2.1. Study Group

This single PH-center, prospective, observational, non-interventional study was conducted during the COVID-19 pandemic. Adult patients with PAH and CTEPH, which were confirmed by right heart catheterization and additional tests according to the current guidelines, were enrolled in the study [4,5]. Patients received specific PH treatment. CTEPH patients with distal disease or persistent pulmonary hypertension were treated with balloon pulmonary angioplasty, even if they had previously undergone a pulmonary endarterectomy. The study began after 14 April 2021, following the approval of the study protocol by the local bioethics committee. The second assessment started after 28 May 2022, when all pandemic-related restrictions had been lifted.

### 2.2. Methods

PAH and CTEPH patients, after giving informed consent and completing appropriate surveys during a routine visit to the PH center were enrolled in the study. The PH center is accredited by the Ministry of Health to provide diagnosis and therapy with pulmonary artery-targeted drugs for patients with PAH and CTEPH, staffed by experts in the field. The study was conducted during the COVID-19 pandemic. All patients completed the questionnaires twice: initially when all restrictions against COVID-19 were in force, and again after 28 March 2022, when restrictions in Poland had been lifted. Patients were examined during a scheduled hospitalization at the center or a scheduled visit to an outpatient clinic.

Patients were ranked on two scales: the Fear of COVID-19 Scale (FCV-19S) and the Hospital Anxiety and Depression Scale (HADS).

The FCV-19S is a psychometric test created by Ahorsu et al. in 2020 to measure the fear and anxiety reaction to the COVID-19 pandemic [15]. The test has been translated into various languages, validated in many countries, and widely used in clinics. It consists of 7 items with answers on a scale from 1 (strongly disagree) to 5 (strongly agree). Scores range from 7 to 35 points, with higher scores indicating greater fear of COVID-19 [15]. FCV-19S has been translated into Polish and validated for the Polish population [16,17].

The HADS reflects the general level of anxiety and depression. It consists of 16 items, 7 of which reflect anxiety, 7 depression, and the last 2 pertain to nervousness. Each item has 4 possible answers, with 0 to 21 points obtainable for each subscale of anxiety or depression [18]. A cut-off value of 8 or more in the HADS anxiety section (HADS-A) or the HADS depression section (HADS-D) can indicate anxious or depressed patients. A cut-off value of 11 or more in the HADS-A or HADS-D section defines patients with severe anxiety or depression [18]. The HADS has been translated into the Polish language and validated for the Polish population [19]. We used both the FCV-19S and HADS in our previous study to measure mental problems in PAH and CTEPH patients during the COVID-19 pandemic [2].

Patients’ demographic characteristics and general information about their disease, treatment and conditions were obtained from their medical records. All participants gave informed written consent to participate in the study. The study protocol was approved by the Bioethics Committee of the Centre of Postgraduate Medical Education in accordance with the Declaration of Helsinki (number KBE 23/2021, date of approval: 14 April 2021).

### 2.3. Stastistical Analysis

The statistical analysis was performed using Statistical Analysis Software (Cary, NC, USA), version 9.4. Categorical variables are presented as numbers with percentages, while normally distributed and non-normally distributed continuous variables are presented as mean values with standard deviations and median values with interquartile ranges, respectively. Distributions of continuous variables were assessed using the Shapiro–Wilk test. For distributions of continuous variables, *p* values < 0.05 in the whole group or in subgroups were considered non-normally distributed. Fisher’s exact test was used to compare categorical variables, and Student’s *t*-test or the Mann–Whitney U test was used to compare continuous variables with normal and non-normal distributions, respectively. Differences for dependent samples were calculated using the Wilcoxon signed-rank test. Spearman correlations were calculated to evaluate the association between continuous variables. A *p* value < 0.05 was considered statistically significant.

## 3. Results

### 3.1. Study Group

**Figure 1:**
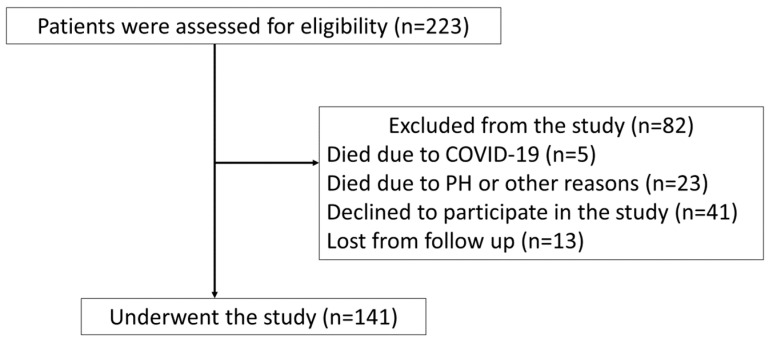
Flowchart of patient enrollment; PH–pulmonary hypertension.

Out of the 223 patients with PAH/CTEPH considered for inclusion in the study, 141 were included for further analysis (Figure 1).

A total of 141 patients were included in the study. Most were female (88; 62%) and had PAH (90; 64%). The patients’ median age was 60 (range 42–72) years. About 78% of the patients were vaccinated against SARS-CoV-2, and 18% had a history of COVID-19. CTEPH patients were older and more often male compared to PAH patients. The baseline characteristics of the study group are presented in Table 1.

### 3.2. Fear of COVID-19, Anxiety, and Depression during the Pandemic

During the first assessment when COVID-19 restrictions were present, the median (IQR) score on the Fear of COVID-19 Scale was 18 (12–23) points. Approximately 26% of patients had excessive anxiety and 16% had depression during this time. Table 2 shows the manifestation of fear of COVID-19, anxiety, and depression in PAH/CTEPH patients at baseline.

Between the baseline and follow-up, 461 (383–629) days passed. The second assessment took place during a routine visit to the PH center after 28 March 2022, when no major pandemic-related restrictions were still in place in Poland. In the study group, the acute negative reaction to COVID-19 had decreased. The FCV-19S scores decreased significantly in the entire study group from 18 (12–23) to 14 (9–21, *p* < 0.001); in the subgroup of patients with PAH from 19 (12–23) to 14 (9–21, *p* < 0.001); and in the subgroup of patients with CTEPH from 17 (11–21) to 14 (8–21, *p* = 0.04). The levels of generalized anxiety and depression did not change (Table 3). Anxiety measured with the HADS-A in the entire study group was 5 (3–8) points at the initial visit and 5 (2–8) points at the follow-up visit (*p* = 0.34). Depression measured with the HADS-D was 3 (1–7) points at the initial visit and 3 (0–6) points at the follow-up visit (*p* = 0.39).

**Figure 2:**
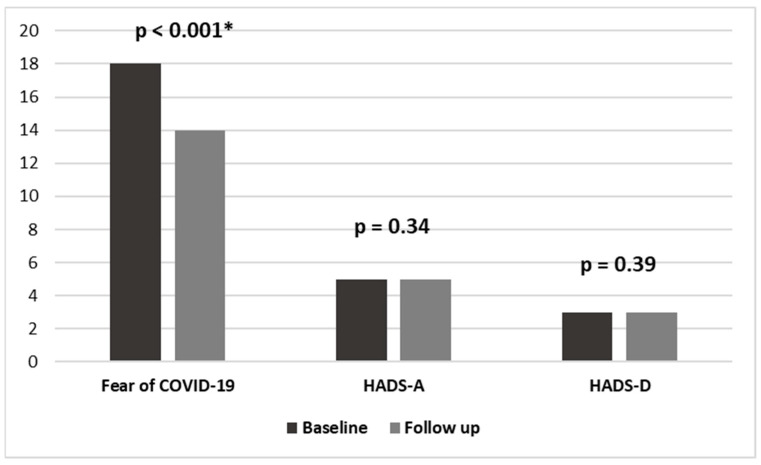
Fear of COVID-19, anxiety (HADS-A), and depression (HADS-D) in the study group between baseline and follow-up (after the lifting of restrictions related to the COVID-19 pandemic); * *p* <0.05.

**Figure 3:**
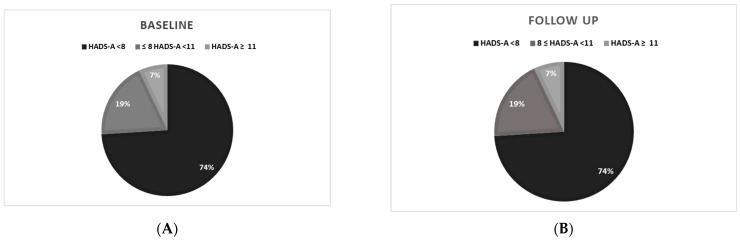
Hospital Anxiety and Depression Scale results (HADS) in PAH and CTEPH patients: (**A**) during the COVID-19 pandemic; (**B**) after removing COVID-19 restrictions; HADS-A (anxiety subscale): HADS-A < 8 normal value, 8 ≤ HADS-A <11 moderate anxiety (anxiety suspected), HADS-A ≥ 11 severe anxiety (anxiety probable).

**Figure 4:**
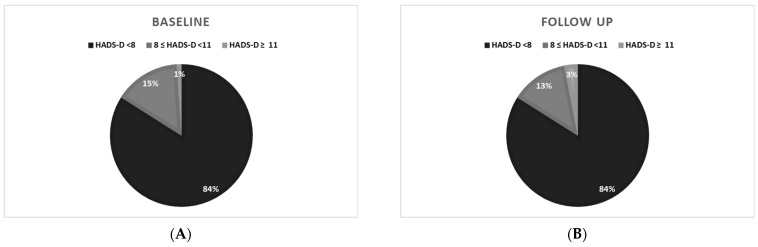
Hospital Anxiety and Depression Scale results (HADS) in PAH and CTEPH patients: (**A**) during the COVID-19 pandemic; (**B**) after removing COVID-19 restrictions; HADS-D (depression subscale): HADS-D < 8 normal value, 8 ≤ HADS-D < 11, moderate depression (depression suspected), HADS-D ≥ 11 severe depression (depression probable).

At follow-up, 26% of PAH/CTEPH patients still felt excessive general anxiety, while 16% were depressed. The detailed distribution of scores on the Fear of COVID-19 Scale is shown in Figure 2, on HADS-A in Figure 2 and Figure 3, and on HADS-D in Figure 2 and Figure 4.

HADS-D, a depression subscale, correlated with PH severity parameters at the beginning of the study, being positively correlated with WHO functional class (rho 0.28; *p* < 0.001) and N-terminal B-type natriuretic propeptide level (NTproBNP, rho = 0.20; *p* = 0.01), and negatively correlated with six-minute walk distance (6 MWD, rho= −0.35; *p* < 0.001). The HADS-D showed no correlation with the FCV-19S (rho = 0.13; *p* = 0.09). The HADS-A, an anxiety subscale showed a positive correlation with FCV-19S (rho 0.26; *p* = 0.002), but not with the PH severity parameters WHO functional class (rho = 0.13; *p* = 0.1), NTproBNP level (rho = 0.06; *p* = 0.4), or 6 MWT (rho= −0.17; *p* = 0.05).

## 4. Discussion

PAH and CTEPH are life-threatening diseases that significantly affect various aspects of a patient’s life. Patients often feel socially isolated, have less opportunities for paid employment, can only have limited contact with friends, cannot travel far, and cannot fully partake in social activities. Consequently, they are forced to reorganize their lives and this also impacts their relatives [20]. Mental health problems are more frequently diagnosed in PH patients than in the general population [21]. Anxiety and depression decrease the quality of life of PAH/CTEPH patients and impair their ability to collaborate with PH centers [11,12,13,14].

In our study, 26% of PAH/CTEPH patients experienced excessive anxiety, and 16% experienced excessive depression. These rates are significantly higher than those in the general population, where the prevalence of depressive disorders was 3% and generalized anxiety disorder was 1.1% several years before the COVID-19 pandemic [22]. Our findings are consistent with those from other PH centers. In a study conducted before the pandemic using the HADS, the prevalence of anxiety and depression was high in PH patients (24% for anxiety and 21% for depression) [11]. Other studies conducted with different psychometric tools, either before the SARS-CoV-2 pandemic or spanning the pandemic, reported high prevalence rates of anxiety (9% to 51%) and depression (7.5% to 56%) in PH patients [12,13,14,20]. Despite the high prevalence, only 13% of depressed or anxious patients in our study received appropriate support and treatment. This may be because PAH and CTEPH patients are typically treated by cardiologists and pulmonologists, who may have limited experience in detecting psychiatric disorders. Despite recommendations, psychosocial support is not available at all PH centers and anxiety–depressive disorders are not actively detected and treated [4,5]. Psychotherapy is the first step to reducing anxiety, while a combination of psychotherapy and drugs is the second step. Pharmacological treatment is the first step to reducing depression and should be applied to PH patients [23].

The first case of coronavirus infection in Poland was recorded on 4 March 2020. From 20 March 2020 to 15 May 2022, the Polish Government, following the Ministry of Health’s directives, imposed a state of emergency, with varying restrictions and periodic lockdowns. Pandemic-related restrictions in Poland changed over time depending on the number of COVID-19 cases and the overall status of the pandemic. The restrictions aimed to reduce the spread of the virus and included the obligation to cover the nose and mouth with a mask; mandatory quarantine and isolation by those who were infected or those who had had contact with a person infected with the SARS-CoV-2 virus; the restriction of movement and limits on the number of people in confined spaces, etc.

In accordance with the Decree of the Council of Ministers on the establishment of certain restrictions, orders, and prohibitions in connection with the outbreak of the epidemic, as of 28 March 2022 all pandemic-related restrictions were lifted [24].

During the lockdown, PH patients were more likely to experience sleep disorders, reduced physical activity, and poorer mental well-being compared to healthy individuals of a similar age [21]. The pandemic revealed many triggers of the stress reaction. PH patients and their caregivers were concerned about delayed contact with healthcare providers, isolation, specific treatment interruptions, and delayed disease exacerbation detection [25,26,27]. As the pandemic continued, it turned out that SARS-CoV-2 infection is associated with a higher risk of death among patients with PAH/CTEPH compared to the general population [28,29,30,31]. Some patients, after undergoing treatment for mild COVID-19 at home, reported symptoms suggesting long COVID syndrome [32]. These circumstances may have predisposed these patients to a greater risk of mental disorders, including anxiety and depression.

FCV-19S is a psychometric tool developed at the beginning of the COVID-19 pandemic to assess emotional reaction to the pandemic, measuring acute distress, anxiety, depression, post-traumatic disorder, specific phobias, and mental instability [15]. When all pandemic-related restrictions were in place in Poland, PAH/CTEPH patients’ fear of COVID-19 was higher compared to the general population, similar to that of patients with other life-threatening diseases such as cancer [2,17,33]. In the study group, the acute reaction related to the pandemic during this follow-up period, as measured by FCV-19S, significantly decreased. This might be due to the activation of various adaptive processes in response to stress as well as to sufficient support from the patients’ families and medical staff [34].

Only one study evaluating the impact of the COVID-19 pandemic on psychiatric disorders in patients with PAH is available in the literature [3]. Park et al. assessed anxiety and depression levels among PAH patients during the first year of the pandemic at two German referral centers [3]. The prevalence of anxiety (measured with the HADS-A) and depression (measured with the HADS-D) were assessed at the beginning of the pandemic and again after 232 days. At the beginning of the study the prevalence of anxiety in the study group was 34%, while depression was 23%. These numbers did not change significantly during the follow-up [3]. Both observations were during the first year of the pandemic, when SARS-CoV-2 infection rates were high, and significant pandemic-related restrictions were in place. The authors concluded that the pandemic did not significantly affect the mental health of PAH patients. The results of our study lead to a similar conclusion, though they involve a much longer follow-up period and concern a different period of the pandemic. Patients with PAH and CTEPH showed only limited changes in anxiety and depression levels, despite severe stress reactions to the COVID-19 pandemic. Anxiety and depression levels at follow-up, when pandemic restrictions relaxed, remained as high as at baseline (26% for anxiety and 16% for depression). Some patients moved from the depression-suspected group to the depression-probable group, but no patient’s HADS-A or HADS-D score changed from unsuspicious to probable.

It can be hypothesized that the serious, life-threatening nature of PAH and CTEPH correlates with an increased risk of chronic anxiety and depression. The pandemic triggered an acute stress response, but patients were more afraid of losing their lives due to severe PH than due to COVID-19. Therefore, anxiety and depression levels among patients with PAH/CTEPH were high regardless of the pandemic’s timing and the severity of the restrictions, but fear of COVID-19 decreased. Thus, PH patients with PH may need additional psychological support or pharmacological treatment to reduce anxiety and depressive disorders.

Our study had some limitations. One significant limitation is the lack of data on anxiety and depression before or after the COVID-19 pandemic in the study group. In the present study, we only compared changes in anxiety and depression at two pandemic time points: during and after the lifting of COVID-19-related restrictions. The restrictions varied in intensity and nature throughout the pandemic, depending on Poland’s epidemiological situation. The study group was relatively small. To assess anxiety and depression, we used the HADS, which has not been previously validated in PAH/CTEPH patients. A meta-analysis of different populations proved that the HADS is good for evaluating anxiety and depression in general populations and various patient groups, including somatic, psychiatric, and primary care patients [35]. Moreover, the HADS was used in our previous study assessing the mental health of PAH/CTEPH patients during the second year of the pandemic and in other studies on mental health and PH patients [3,11]. We did not collect additional data on factors that might have impacted the obtained results on anxiety or fear, such as education level, place of residence, marital status, addiction, and media habits.

## 5. Conclusions

In conclusion, the COVID-19 pandemic triggered an acute emotional response, but had little effect on the severity of anxiety and depression in PAH/CTEPH patients after the COVID-19 restrictions were relaxed. This suggests that factors beyond the COVID-19 pandemic may contribute to the development of mental illness in PAH/CTEPH patients. PAH/CTEPH patients may need active detection and additional psychological support or pharmacological treatment to reduce anxiety and depressive disorders.

## Figures and Tables

**Table 1:** Baseline characteristics of the study group patients according to the pulmonary hypertension type.

	Total Study Group n (%) or Mean (SD)	PAH n (%) or Mean (SD)	CTEPH n (%) or Mean (SD)	PAH vs. CTEPH
Number of patients	141 (100%)	90 (64%)	51 (36%)	
Females/males	88/53 (62%/38%)	69/21 (77%/33%)	19/32 (37%/63%)	<0.001 *
Age, years	60 (42–72)	57 (41–69)	68 (48–78)	0.005 *
Duration of disease, years	5.7 (2.1–9.8)	5.8 (2.3–9.8)	3.8 (1.2–9.8)	0.22
PAH patients				
• Idiopathic PAH		54 (60%)		
• PAH associated with CHD		9 (10%)		
• PAH associated with CTD		17 (19%)		
• Heritable PAH		6 (7%)		
• PAH porto-pulmonary		4 (4%)		
PAH monotherapy		18 (23%)		
PAH double combination therapy		33 (42%)		
PAH triple combination therapy		27 (34%)		
CTEPH-BPA			42 (82%)	
CTEPH-PEA			12 (24%)	
CTEPH PAH-like therapy (riociguat or sildenafil)			45 (88%)	
WHO functional class	2.4 (±0.6)	2.5 (±0.6)	2.3 (±0.7)	0.4
WHO FC 1	9 (6.4%)	2 (2%)	6 (12%)	
WHO FC 2	71 (50%)	47 (52%)	22 (43%)	
WHO FC 3	59 (42%)	37 (41%)	23 (45%)	
WHO FC 4	2 (1.4%)	4 (4.4%)	0	
6 MWD, m	457 (330–540)	459 (333–542)	453 (330–523)	0.41
NTproBNP (pg/mL)	237 (99–810)	249 (107–673)	201 (89–994)	0.61
1-year risk of death due to PH, points #	1.6 (1.0–2.6)	1.6 (1.0–2.5)	1.6 (1.0–2.6)	0.86
COVID-19 vaccination	107 (76%)	68 (76%)	39 (76%)	1.0
History of COVID-19	26 (18%)	15 (17%)	11 (22%)	0.5
History of depression or anxiolytic treatment	19 (13%)	13 (14%)	6 (12%)	0.8
Concomitant disease	93 (66%)	56 (62%)	37 (73%)	0.27
Arterial hypertension	62 (44%)	36 (40%)	26 (51%)	0.22
Diabetes	18 (13%)	13 (14%)	5 (10%)	0.60
Coronary artery disease	23 (16%)	10 (11%)	4 (8%)	0.77
COPD	14 (9.9%)	13 (14%)	10 (20%)	0.48
Neoplasm	16 (11%)	8 (9%)	8 (16%)	0.27
Obesity, BMI ≥ 30 kg/m^2^	41 (29%)	24 (27%)	17 (33%)	0.44

PH—pulmonary hypertension; PAH—pulmonary arterial hypertension; PAH-CHD—pulmonary arterial hypertension related to congenital heart disease; IPAH—idiopathic pulmonary hypertension; PAH-CTD—pulmonary arterial hypertension associated with connective tissue disease; PAH-porto-pulmonary—pulmonary arterial hypertension associated with portal hypertension; CTEPH—chronic thromboembolic pulmonary hypertension; BPA—balloon pulmonary angioplasty; PEA—pulmonary endarterectomy; COPD—chronic obstructive pulmonary disease; WHO FC—World Health Organization Functional Class; # simplified risk-assessment tool COMPERA 2.0—Comparative, Prospective Registry of Newly Initiated Therapies for PH; 6 MWD—six-minute walk distance; NTproBNP—N-terminal pro B-type natriuretic peptide. *p* < 0.05 *.

**Table 2:** Baseline results of FCV-19S, HADS-A, and HADS-D in the study group.

	All Patients n (%); Median (IQR) or Mean (SD) n = 141	PAH n (%); Median (IQR) or Mean (SD) n = 90	CTEPH n (%); Median (IQR) or Mean (SD) n = 51	PAH vs. CTEPH
Fear of COVID-19, points	18 (12–23)	19 (12–23)	17 (11–21)	0.32
HADS-A, points	5 (3–8)	5 (3–8)	5 (2–8)	0.54
HADS-D, points	3 (1–7)	2 (1–7)	4 (0–7)	0.64
HADS-A ≥ 8 points	37 (26%)	24 (27%)	13 (25%)	0.69
HADS-A ≥ 11 points	10 (7%)	6 (6.7%)	4 (7.8%)	1.0
HADS-D ≥ 8 points	23 (16%)	16 (18%)	7 (14%)	0.64
HADS-D ≥ 11 points	4 (2.8%)	2 (2%)	0	0.54

FCV-19S—Fear of COVID-19 Scale; HADS—Hospital Anxiety and Depression Scale; HADS-A—Hospital Anxiety and Depression Scale—anxiety subscale; HADS-D—Hospital Anxiety and Depression Scale—depression subscale; PAH—pulmonary arterial hypertension; CTEPH—chronic thromboembolic pulmonary hypertension.

**Table 3:** Changes in fear of COVID-19 (FOC), anxiety (HADS-A), and depression (HADS-D) in the study group between baseline and follow-up (after the lifting of restrictions related to the COVID-19 pandemic).

	All n = 141	PAH (n = 90)	CTEPH (n = 51)	P (PAH vs. CTEPH)
∆ FCV-19S	−4 (−7–0), *p* < 0.001 *	−4 (−7–0), *p* < 0.001 *	−3 (−7–2), *p* = 0.04 *	0.68
∆ HADS-A ∆ HADS-D	−2 (−4–0), *p* = 0.34 * 0 (−2–1), *p* = 0.39 *	0 (−2–1), *p* = 0.22 * 0 (−1–1), *p* = 0.69 *	0 (−2–2), *p* = 0.99 * 0 (−2–1), *p* = 0.37 *	0.36 0.71

FCV-19S—Fear of COVID-19 Scale; HADS—Hospital Anxiety and Depression Scale; HADS-A—Hospital Anxiety and Depression Scale—anxiety subscale; HADS-D—Hospital Anxiety and Depression Scale—depression subscale; PAH—pulmonary arterial hypertension; CTEPH—chronic thromboembolic pulmonary hypertension; * *p* value for change from baseline.

## Data Availability

Data are contained within the article.

## References

[1] Farooq S., Tunmore J., Wajid Ali M., Ayub M. (2021). Suicide, self-harm and suicidal ideation during COVID-19: A systematic review. Psychiatry Res..

[2] Wieteska-Milek M., Szmit S., Florczyk M., Kusmierczyk-Droszcz B., Ryczek R., Dzienisiewicz M., Torbicki A., Kurzyna M. (2021). Fear of COVID-19, Anxiety and Depression in Patients with Pulmonary Arterial Hypertension and Chronic Thromboembolic Pulmonary Hypertension during the Pandemic. J. Clin. Med..

[3] Park D.H., Fuge J., Meltendorf T., Kahl K.G., Richter M.J., Gall H., Ghofrani H.A., Kamp J.C., Hoeper M.M., Olsson K.M. (2021). Impact of SARS-CoV-2-Pandemic on Mental Disorders and Quality of Life in Patients with Pulmonary Arterial Hypertension. Front. Psychiatry.

[4] Humbert M., Kovacs G., Hoeper M.M., Badagliacca R., Berger R.M.F., Brida M., Carlsen J., Coats A.J.S., Escribano-Subias P., Ferrari P. (2022). 2022 ESC/ERS Guidelines for the diagnosis and treatment of pulmonary hypertension. Eur. Respir. J..

[5] Galie N., Humbert M., Vachiery J.L., Gibbs S., Lang I., Torbicki A., Simonneau G., Peacock A., Vonk Noordegraaf A., Beghetti M. (2016). 2015 ESC/ERS Guidelines for the diagnosis and treatment of pulmonary hypertension: The Joint Task Force for the Diagnosis and Treatment of Pulmonary Hypertension of the European Society of Cardiology (ESC) and the European Respiratory Society (ERS): Endorsed by: Association for European Paediatric and Congenital Cardiology (AEPC), International Society for Heart and Lung Transplantation (ISHLT). Eur. Heart J..

[6] Kopec G., Dzikowska-Diduch O., Mroczek E., Mularek-Kubzdela T., Chrzanowski L., Skoczylas I., Tomaszewski M., Peregud-Pogorzelska M., Karasek D., Lewicka E. (2021). Characteristics and outcomes of patients with chronic thromboembolic pulmonary hypertension in the era of modern therapeutic approaches: Data from the Polish multicenter registry (BNP-PL). Ther. Adv. Chronic Dis..

[7] Darocha S., Roik M., Kopec G., Araszkiewicz A., Furdal M., Lewandowski M., Jachec W., Grabka M., Banaszkiewicz M., Pietrasik A. (2022). Balloon pulmonary angioplasty in chronic thromboembolic pulmonary hypertension: A multicentre registry. EuroIntervention.

[8] Ivanauskiene T., Cesna S., Grigoniene E., Gumbiene L., Daubaraite A., Ivanauskaite K., Glaveckaite S. (2024). Balloon Pulmonary Angioplasty for Inoperable Chronic Thromboembolic Pulmonary Hypertension: Insights from a Pilot Low-Volume Centre Study and a Comparative Analysis with Other Centres. Medicina.

[9] Kopec G., Kurzyna M., Mroczek E., Chrzanowski L., Mularek-Kubzdela T., Skoczylas I., Kusmierczyk B., Pruszczyk P., Blaszczak P., Lewicka E. (2020). Characterization of Patients with Pulmonary Arterial Hypertension: Data from the Polish Registry of Pulmonary Hypertension (BNP-PL). J. Clin. Med..

[10] Kurzyna M., Zylkowska J., Fijalkowska A., Florczyk M., Wieteska M., Kacprzak A., Burakowski J., Szturmowicz M., Wawrzynska L., Torbicki A. (2008). Characteristics and prognosis of patients with decompensated right ventricular failure during the course of pulmonary hypertension. Pol. Heart J. (Kardiol. Pol.).

[11] Somaini G., Hasler E.D., Saxer S., Huber L.C., Lichtblau M., Speich R., Bloch K.E., Ulrich S. (2016). Prevalence of Anxiety and Depression in Pulmonary Hypertension and Changes during Therapy. Respiration.

[12] Pfeuffer E., Krannich H., Halank M., Wilkens H., Kolb P., Jany B., Held M. (2017). Anxiety, Depression, and Health-Related QOL in Patients Diagnosed with PAH or CTEPH. Lung.

[13] Olsson K.M., Meltendorf T., Fuge J., Kamp J.C., Park D.H., Richter M.J., Gall H., Ghofrani H.A., Ferrari P., Schmiedel R. (2021). Prevalence of Mental Disorders and Impact on Quality of Life in Patients With Pulmonary Arterial Hypertension. Front. Psychiatry.

[14] Dering M.R., Lepsy N., Fuge J., Meltendorf T., Hoeper M.M., Heitland I., Kamp J.C., Park D.H., Richter M.J., Gall H. (2022). Prevalence of Mental Disorders in Patients With Chronic Thromboembolic Pulmonary Hypertension. Front. Psychiatry.

[15] Ahorsu D.K., Lin C.Y., Imani V., Saffari M., Griffiths M.D., Pakpour A.H. (2020). The Fear of COVID-19 Scale: Development and Initial Validation. Int. J. Ment. Health Addict..

[16] Pisula E., Nowakowska I. (2020). Skala Lęku Przed Koronawirusem FCV-19S (Ahorsu i in., 2020)—Polskie tłumaczenie.

[17] Pilch I., Kurasz Z., Turska-Kawa A. (2021). Experiencing fear during the pandemic: Validation of the fear of COVID-19 scale in Polish. PeerJ.

[18] Zigmond A.S., Snaith R.P. (1983). The hospital anxiety and depression scale. Acta Psychiatr. Scand..

[19] Wichowicz H.M., Wieczorek D. (2011). Screening post-stroke depression using the Hospital Anxiety and Depression Scale. Psychiatr. Pol..

[20] White J., Hopkins R.O., Glissmeyer E.W., Kitterman N., Elliott C.G. (2006). Cognitive, emotional, and quality of life outcomes in patients with pulmonary arterial hypertension. Respir. Res..

[21] Dobler C.L., Kruger B., Strahler J., Weyh C., Gebhardt K., Tello K., Ghofrani H.A., Sommer N., Gall H., Richter M.J. (2020). Physical Activity and Mental Health of Patients with Pulmonary Hypertension during the COVID-19 Pandemic. J. Clin. Med..

[22] Kiejna A., Adamowski T., Piotrowski P., Moskalewicz J., Wojtyniak B., Swiatkiewicz G., Stokwiszewski J., Kantorska-Janiec M., Zagdanska M., Kessler R. (2015). “Epidemiology of mental disorders and access to mental health care. EZOP—Poland”—Research methodology. Psychiatr. Pol..

[23] Pilling S., Mayo-Wilson E., Mavranezouli I., Kew K., Taylor C., Clark D.M., Guideline Development G. (2013). Recognition, assessment and treatment of social anxiety disorder: Summary of NICE guidance. BMJ.

[24] (2022). Coronawirus Information and Recommendation. https://www.gov.pl/web/coronavirus/temporary-limitations.

[25] Kopec G., Tyrka A., Jonas K., Magon W., Waligora M., Stepniewski J., Podolec P. (2020). The coronavirus disease 2019 pandemic prevents patients with pulmonary hypertension from seeking medical help. Pol. Heart J. (Kardiol. Pol.).

[26] Kwiatkowska J., Meyer-Szary J., Mazurek-Kula A., Zuk M., Migdal A., Kusa J., Skiba E., Zygielo K., Przetocka K., Kordon Z. (2021). The Impact of COVID-19 Pandemic on Children with Pulmonary Arterial Hypertension. Parental Anxiety and Attitudes. Follow-Up Data from the Polish Registry of Pulmonary Hypertension (BNP-PL). J. Clin. Med..

[27] Godinas L., Iyer K., Meszaros G., Quarck R., Escribano-Subias P., Vonk Noordegraaf A., Jansa P., D’Alto M., Luknar M., Milutinov Ilic S. (2021). PH CARE COVID survey: An international patient survey on the care for pulmonary hypertension patients during the early phase of the COVID-19 pandemic. Orphanet J. Rare Dis..

[28] Belge C., Quarck R., Godinas L., Montani D., Escribano Subias P., Vachiery J.L., Nashat H., Pepke-Zaba J., Humbert M., Delcroix M. (2020). COVID-19 in pulmonary arterial hypertension and chronic thromboembolic pulmonary hypertension: A reference centre survey. ERJ Open Res..

[29] Wieteska-Milek M., Kusmierczyk-Droszcz B., Ryczek R., Szmit S., Florczyk M., Manczak R., Betkier-Lipinska K., Hoffman P., Krzesinski P., Torbicki A. (2023). Outcomes of COVID-19 in patients vaccinated and unvaccinated against SARS-CoV-2 and suffering from pulmonary arterial hypertension and chronic thromboembolic pulmonary hypertension. Pol. Arch. Intern. Med..

[30] Lee J.D., Burger C.D., Delossantos G.B., Grinnan D., Ralph D.D., Rayner S.G., Ryan J.J., Safdar Z., Ventetuolo C.E., Zamanian R.T. (2020). A Survey-based Estimate of COVID-19 Incidence and Outcomes among Patients with Pulmonary Arterial Hypertension or Chronic Thromboembolic Pulmonary Hypertension and Impact on the Process of Care. Ann. Am. Thorac. Soc..

[31] Montani D., Certain M.C., Weatherald J., Jais X., Bulifon S., Noel-Savina E., Nieves A., Renard S., Traclet J., Bouvaist H. (2022). COVID-19 in Patients with Pulmonary Hypertension: A National Prospective Cohort Study. Am. J. Respir. Crit. Care Med..

[32] Wieteska-Milek M., Kusmierczyk-Droszcz B., Betkier-Lipinska K., Szmit S., Florczyk M., Zielinski P., Hoffman P., Krzesinki P., Kurzyna M. (2023). Long COVID syndrome after SARS-CoV-2 survival in patients with pulmonary arterial hypertension and chronic thromboembolic pulmonary hypertension. Pulm. Circ..

[33] Sigorski D., Sobczuk P., Osmola M., Kuc K., Walerzak A., Wilk M., Ciszewski T., Kopec S., Hryn K., Rutkowski P. (2020). Impact of COVID-19 on anxiety levels among patients with cancer actively treated with systemic therapy. ESMO Open.

[34] Heitzman J. (2020). Impact of COVID-19 pandemic on mental health. Psychiatr. Pol..

[35] Bjelland I., Dahl A.A., Haug T.T., Neckelmann D. (2002). The validity of the Hospital Anxiety and Depression Scale. An updated literature review. J. Psychosom. Res..

